# Should we use large language models to perform systematic reviews?

**DOI:** 10.1093/bib/bbaf631.059

**Published:** 2025-12-12

**Authors:** TH Chan, SH Jin, Mun Kay Ho, Joshua W K Ho

**Affiliations:** La Salle College, Hong Kong SAR, China; La Salle College, Hong Kong SAR, China; School of Biomedical Sciences, Li Ka Shing Faculty of Medicine, The University of Hong Kong, Hong Kong SAR, China; School of Biomedical Sciences, Li Ka Shing Faculty of Medicine, The University of Hong Kong, Hong Kong SAR, China

## Abstract

**Background:**

Large language models (LLMs) have shown promising performances in literature search, screening of full-text scientific articles, data extraction, and produce a written summary. LLMs have a strong potential in automating the process of conducting systematic reviews. Nonetheless, conducting systematic reviews is also a training exercise for critical appraisal of scientific evidence and synthesis of scientific claims. Overreliance on LLMs may erode comprehension and analytical skills in reading scientific articles over time, leading to cognitive offloading. Other risks may involve hallucination and responsibility problem.

**Methods:**

To address both opportunities and limitations of LLMs when applied in systematic reviews, the LLM-Augmented Systematic Evidence Review (LASER) framework is proposed.

**Results:**

The framework recommends different degree of LLM usage in different steps in the systematic review process to promote efficiency, ensure accuracy, and prevent cognitive offloading of the researcher involved. In this framework, the specific degree of LLM usage may depend on the career stage of the researcher and the accuracy of the LLM.

